# ASSET: Auto-Segmentation of the Seventeen SEgments for Ventricular Tachycardia Ablation in Radiation Therapy

**DOI:** 10.3390/cancers15164062

**Published:** 2023-08-11

**Authors:** Eric Morris, Robert Chin, Trudy Wu, Clayton Smith, Siamak Nejad-Davarani, Minsong Cao

**Affiliations:** 1 Department of Radiation Oncology, Washington University, St. Louis, MO 63110, USA; 2 Department of Radiation Oncology, UCLA Health, Los Angeles, CA 90095, USA; rkchin@mednet.ucla.edu (R.C.); tcwu@mednet.ucla.edu (T.W.); claytonsmith@mednet.ucla.edu (C.S.); 3 Department of Radiation Oncology, University of Miami Miller School of Medicine, Miami, FL 33136, USA; spn717@miami.edu

**Keywords:** radiation therapy, cardiac ablation, ventricular tachycardia, automatic segmentation

## Abstract

**Simple Summary:**

The segmentation of the ventricular tachycardia target is complex, error-prone, and time-consuming when performed manually. Currently, target generation for ventricular tachycardia is performed manually through viewing electro-anatomic mapping which is incredibly error-prone due to the current lack of a method to directly register electro-anatomic data to radiation therapy planning images. This work presents a novel model to automatically provide the 17 segments of the left ventricle as defined by the American Heart Association on any imaging modality for radiation therapy planning. This is completed through the use of principal component analysis. These segments can then be used as an aid for radiation therapy planning for physicians and physicists in numerous ways. Namely, by offering significant improvements to target generation consistency and time-saving measures which in turn offers strong potential for widespread application to institutions conducting radio-ablation of the left ventricular myocardium.

**Abstract:**

There has been a recent effort to treat high-risk ventricular tachycardia (VT) patients through radio-ablation. However, manual segmentation of the VT target is complex and time-consuming. This work introduces ASSET, or Auto-segmentation of the Seventeen SEgments for Tachycardia ablation, to aid in radiation therapy (RT) planning. ASSET was retrospectively applied to CTs for 26 thoracic RT patients (13 undergoing VT ablation). The physician-defined parasternal long-axis of the left ventricle (LV) and the axes generated from principal component analysis (PCA) were compared using mean distance to agreement (MDA) and angle of separation. The manually selected right ventricle insertion point and LVs were used to apply the ASSET model to automatically generate the 17 segments of the LV myocardium (LVM). Physician-defined parasternal long-axis differed from PCA by 1.2 ± 0.3 mm MDA and 6.9 ± 0.7 degrees. Segments differed by 0.69 ± 0.29 mm MDA and 0.89 ± 0.03 Dice similarity coefficient. Running ASSET takes <5 min where manual segmentation took >2 h/patient. Agreement between ASSET and expert contours was comparable to inter-observer variability. Qualitative scoring conducted by three experts revealed automatically generated segmentations were clinically useable as-is. ASSET offers efficient and reliable automatic segmentations for the 17 segments of the LVM for target generation in RT planning.

## 1. Introduction

Ventricular tachycardia (VT) is a major cardiac arrhythmia that has shown to be life threatening. For example, in the US, deaths from VT are equal to the deaths from the top four cancers combined. VT may arise from coronary artery disease (CAD) (e.g., scar-related VT) or other structural cardiomyopathies (e.g., non-ischemic dilated cardiomyopathy (DCM)) and is life-threatening as it can degenerate into ventricular fibrillation (Vfib) and cardiac arrest [1].

Current treatments of VT involve the placement of a cardiovascular implantable electronic device (CIED) that monitors the heart’s rhythm and can detect and deliver an electric shock in an attempt to correct an abnormal rhythm [2]. Antiarrhythmic drugs (AADs) and radiofrequency catheter ablation (RFA) can also significantly decrease the ventricular arrhythmia burden. However, RFA may be associated with significant procedural risks for high-risk patients and has an average success rate near 70% [2]. Furthermore, many VT patients exhibit RFA-refractory disease and require multiple procedures [3].

Stereotactic body radiation therapy (SBRT) is a form of hypo-fractionated radiation therapy that makes use of image guidance to deliver focused high-dose ionizing radiation beams to a precise location in the body while minimizing the dose to surrounding healthy normal tissue. Recently, SBRT has been employed at multiple institutions as a non-invasive treatment option to target the arrhythmogenic substrate for patients with VT who are refractory to AADs or are not candidates for RFA. Contra-indications for endocardial RFA include single peripheral lesions of 5 cm or greater, more than three lesions where any are greater than 3 cm, and patients who are poor surgical candidates due to multiple comorbidities etc. [4]. Numerous recent studies have employed SBRT treatments for non-invasive radio-ablation for arrhythmias [5,6], cardiac fibromas [7], and other cardiac indications [1]. In a study by Cuculich et al., the treatment of VT in a single fraction of 25 Gy using SBRT on TrueBeam (Varian Medical Systems, Palo Alto, CA, USA) for five patients resulted in a significant reduction in total cardiac episodes [5]. Overall, the use of SBRT for cardiac ablation of VT has shown favorable outcomes for patients with limited alternative options [5,8,9,10].

Although SBRT has been presented as a promising treatment alternative to conventional therapies for VT, defining the treatment target in this setting remains challenging because of the difficulties in integrating electrophysiological information (e.g., 12-lead surface electrocardiogram (ECG) and electro-anatomic mapping (EAM)) to radiation treatment planning images. This issue is well known and is a significant obstacle to the much wider utilization of this technique. One promising approach has been to define the target using the American Heart Association (AHA) standard 17-segment contour model [11]. The 17-segment model from the AHA has an anatomical basis, the segments can be reasonably identified using echocardiographic landmarks, and it has been used and validated in several multicenter co-operative studies [12]. However, defining these 17-segment contours manually on a radiation therapy treatment planning computed tomography (CT) dataset can be as difficult, time-consuming, and error-prone with large inter-observer variability as defining the target itself. Thus, creating an easy and reliable means of automatically and consistently generating the 17-segment contours on images for radiation therapy is necessary.

In this work, we have created an automated segmentation tool called ASSET (Auto-segmentation of the Seventeen SEgments for ventricular Tachycardia ablation) to be easily implemented into clinical practice to facilitate target delineation for SBRT treatment of VT. Once the left ventricle (LV) and the right ventricle (RV) insertion points are manually defined by a physician on the planning CT images, a patient-specific 17-segment myocardial contour model will be automatically generated according to the AHA standard. ASSET is a patient-specific 17-segment contour model that localizes the substrate targets obtained from electrophysiological systems to assist with the final planning target volume delineation for radiation therapy.

This segmentation tool provides valuable assistance for target-volume delineation for treatment of refractory ventricular tachycardia by allowing clear and reliable identification of the target zone, even for novice practitioners. This tool may also be of significant interest to both radiotherapy device manufacturers and purveyors of third-party contouring/treatment planning software.

## 2. Materials and Methods

### 2.1. Patient Population and Treatment

Twenty-six patients were consented to an Institutional Review Board-approved study. Thirteen of these patients were treated with SBRT for VT as they previously failed numerous RFA procedures or were considered high-risk patients due to multiple comorbidities [8]. Additionally, these patients were not eligible for other life-saving therapies such as thoracic surgery or cardiac transplantation. Of the 13 single fraction treatment courses, one was prescribed to 15 Gy and three were prescribed to 20 Gy as normal tissue constraints could not be met at a higher target dose. The other nine were prescribed to 25 Gy in a single fraction. Treatments were completed through the use of 2–4 arcs with 6 MV beams and volumetric modulated arc therapy (VMAT) which were delivered on a Novalis Tx Linear Accelerator (Brainlab, Inc., Westchester, IL, USA). Please refer to the reference by Chin et al. for further treatment details [8]. The other 13 patients were retrospectively evaluated in the study as they received thoracic radiation therapy in close proximity to the heart under the presence of a CIED. All patients within the last five years meeting this criteria at our institution were considered and included in the study and the representation of the total population was unbiased.

### 2.2. Patient Imaging 

For all 26 patients in the cohort, free-breathing CTs were acquired with a Siemens SOMATOM CT Scanner (Siemens Healthcare Diagnostics, Los Angeles, CA, USA). The in-plane resolution of the CT was 0.98 mm^2^ for a field of view of 50 cm^2^ and 512 × 512 pixels with a slice thickness of 1.5 mm. An MRI was taken at 1.5 or 3T (Avanto, Siemens Medical Systems, Malvern, PA, USA) with a sequence explicitly established to remove the hyper-intensity artifacts induced by ICDs for myocardial scar delineation [13]. The sequence was a modified wideband late gadolinium enhancement (LGE) MRI imaging technique [13]. The in-plane resolution of the MR ranged from 1.4–1.6 mm^2^ with a slice thickness of 3–4 mm. Lastly, CT and MR datasets were exported and registered in MIM (MIM Inc., Beachwood, OH, USA) for delineation.

### 2.3. Left Ventricular Segmentation

A manual consensus segmentation of the LV was generated for all 26 patients by an expert physician and physicist on the non-contrast planning CT dataset. The expert physician and physicist have been the primary overseers of all institutional cardiac VT clinical treatments for five years. Consensus was defined as generation by the expert physician and then review by the expert physicist. The LVM was then defined by creating an 8 mm inner ring on the LV which is consistent with the methods of Kawel et al. [14]. A standardized margin was used due to insufficient image contrast to consistently resolve the endocardium of the LV. However, users with images that have sufficient contrast to resolve the endocardium of the LV can apply the ASSET model in the same method described herein with no adaptations to the workflow. Lastly, the area containing the mitral valve was then manually removed to create the final conical shape of the LVM.

### 2.4. Definition of 17 Left Ventricular Segments

**Figure 1:**
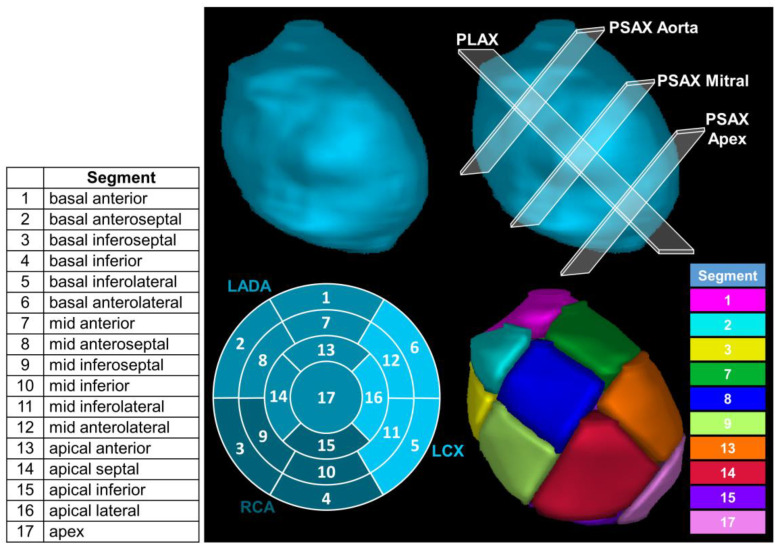
(**Top left**): Three-dimensional segmentation of left ventricle (LV). (**Top right**): The parasternal long-axis (PLAX) is the plane through the longest section of the LV and where the primary principal component goes through. The three parasternal short-axis (PSAX) planes were used to define the apex and three equal regions of equal length down the PLAX (i.e., the basal, mid-cavitary, and apical regions). (**Bottom left**): American Heart Association bulls eye displaying how segments are split. The three coronary artery territories are the left anterior descending artery (LADA), the right coronary artery (RCA), and the left circumflex coronary artery (LCX). (**Bottom right**): Display of the left ventricle split into 17 segments (color key denoting each displayed LV segment in the (**bottom right**)) based on 17-segment American Heart Association model.

The AHA recommends that the LV be divided into 17 individual segments as it allows for precise localization and provides an adequate sampling of the coronary distribution [11]. This can be seen by the bullseye diagram in the bottom left of Figure 1. Additionally, having a standardized and consistent model optimizes both the inter- and intra-modality evaluations that are completed for diagnosis and treatment planning. Our definitions of the 17 segments of the LV follow published consensus guidelines [15]. The work by Brownstein et al. [16] provides a suggested step-by-step approach on how to orient and delineate the 17 segments of the LV manually based on the utilized AHA model.

In short, to define the 17 segments of the LV, the LV is split into four regions (i.e., the apex, apical, mid, and basal regions) using three planes along the parasternal short-axis (PSAX, shown in the top right of Figure 1). Not considering the apex of the heart, the apical, mid, and basal regions are split into equivalent lengths along the axis of the heart. Further, the mid and basal regions are subdivided into six segments of 60° each and the apical region is subdivided into four segments of 90° each. The wall segments are defined based upon internal anatomical landmarks [17] and the myocardium is standardized to an 8 mm thickness, yielding the 17 segments listed in Figure 1 according to the AHA [11].

### 2.5. Applying the ASSET Model

To automatically generate the 17 segments of the LV defined in Section 2.4, the axis through the LV that runs the length of its longest portion (i.e., parasternal long-axis) needs to be defined. The expert physician defined this axis by rotating the dataset in the axial and sagittal view and then selecting two points; the first at the most basal and the second at the most apical slice. The physician-defined points were generated through an in-house workflow generated in MIM (MIM Inc., Beachwood, OH, USA). Additional details on this workflow can be found in Section 3.1. The line generated from these two physician-defined points was then compared to an automatically generated axis accomplished through the use of principal component analysis (PCA). PCA is a mathematical method of ordination designed to simplify and visualize multivariate data [18]. The greatest variability in the dataset can be described by the first eigenvector (i.e., major axis regression) [18]. This line of major axis regression is also commonly referred to as the first principal component. Implementation of PCA was completed in the MATLAB programming environment (Mathworks Inc, Natick, MA, USA) and accessed through an additional integrated MIM workflow. For further details on how the resulting segments were generated after creating the first principal component or physician-drawn parasternal long-axis (PLAX), please refer to Section 3.1.

Morphological closing was used for post-processing after the endocardial and epicardial surfaces of the LV were divided into 17 segments. Morphological closing is a post-processing technique to fuse narrow breaks and eliminate small holes. The process of morphologically closing a segmentation involves a dilation followed by an erosion with the same structuring element for both operations [19]. The structuring element used for morphological closing post-processing of each of the 17 segments was implemented in MIM.

This study was performed using the Windows 10 operation system. The system is 64-bit and equipped with an AMD Ryzen 7 1800X eight-core processor at 3.2 GHz and 16 GB of memory. The employed graphics processing unit was an NVIDIA GeForce GTX 1080 Ti.

### 2.6. Data Extraction

To evaluate inter-observer variability, 10 patients were randomly selected to have each of the 17 segments manually segmented by three radiation oncologists. Manual segmentations of the 10 patients were then compared to the automatically generated segmentations from the ASSET model. To evaluate the efficiency gain from the ASSET model, the manual segmentation time for the three experts and the time to run the model was recorded. In order to validate the model accuracy, the physician-generated manual PLAX was evaluated against the automatically generated first principal component line. These two lines were evaluated by finding the mean distance between the lines, as well as the angle difference between the lines. The Dice similarity coefficient (DSC) and mean distance to agreement (MDA) were also calculated for each segment between the 17 segments generated with the first principal component and the manual PLAX.

### 2.7. Dosimetric Assessment

For the 13 patients in the cohort that underwent cardiac SBRT, the delivered dose was retrospectively evaluated against the ASSET model segment locations. Treatment notes detailing which LV segments had involved VT were tabulated. Dose was overlaid onto the ASSET model and qualitatively compared to the original treatment note.

### 2.8. Qualitative Analysis

To assess the clinical viability of the ASSET model, each of the 17 segments for 10 patients were qualitatively scored based on the following published five-point scale [20,21]:

(1) Clinically unacceptable
(2) Major modifications required
(3) Moderate modifications required
(4) Minor modifications required
(5) Clinically acceptable

Qualitative scoring was completed by three radiation oncologists. The first of the three physicians was the expert physician mentioned in Section 2.5, the other two physicians were heavily involved in institutional cardiac VT clinical treatments who received formal training from the expert physician.

## 3. Results

### 3.1. Generation of the 17 Segments

**Figure 2:**
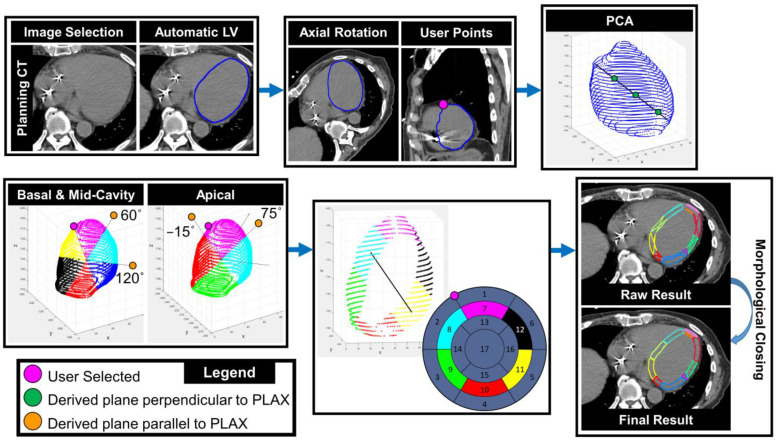
ASSET Workflow. (**Top left**): CT dataset is selected and left ventricle (LV) is manually delineated. (**Top middle**): Image is rotated axially and sagittally to define the right ventricle (RV) insertion point (magenta circle). (**Top right**): Principal component analysis (PCA) is used to identify the parasternal long-axis (PLAX, black line) and the three points to define the three parasternal short axes are derived (green circles). (**Bottom left**): A vector perpendicular to the PLAX was found at 60° and 120° from the user-selected RV insertion point to identify the three planes used for the basal and mid-cavitary regions (2 points at −15° and 75° for the apical region) of the left ventricle myocardium (LVM). (**Bottom middle**): all planes are combined to identify regions for each of the 17 cardiac segments. (**Bottom right**): Morphological closing is used to generate final contours of the 17 segments.

The generated workflow of the ASSET model is outlined in Figure 2 and is described as follows. The dataset to be segmented, either CT or MR, is selected and an LV segmentation is manually generated. An 8 mm ring inside the LV is generated to establish the definition of the LVM. The user then rotates the dataset axially and sagittally to the double barrel view to select the RV insertion point, i.e., the superior point where the right ventricle touches the LVM (Figure 2, magenta circle). The entire process after this point is automatically run through a MATLAB script which is accessed through a MIM workflow which starts with the definition of the first principal component line (Figure 2, black line). Then, the three points to define the three parasternal short axes are automatically derived and defined (Figure 2, green circles). Shown in the bottom left of Figure 2, vectors perpendicular to the PLAX are located at 60° and 120° from the RV insertion point to identify the three planes used for the basal and mid-cavitary regions of the LVM. For the apical region, two points at −15° and 75° are defined perpendicular to the PLAX. Each of the derived planes are combined to identify regions for each of the 17 cardiac segments and the points from the original LVM segmentation are assigned to each region. An example of the points assigned to the mid-cavitary region along with the associated segments on the AHA bullseye diagram is shown in the bottom middle of Figure 2. As the assigned points are only on the endocardial and epicardial surfaces, morphological closing is used to generate final contours of the 17 segments. Due to patients presenting with differing anatomy, the number of segments that appear on an axial slice will vary depending on the angle of the PLAX.

### 3.2. Model Performance

Once the LV is manually segmented and the RV insertion point is defined (~5 minutes), the ASSET model takes less than 5 minutes to run for a total time less than 10 minutes. In comparing the physician-defined PLAX to the first principal component across the 26 patients, it was found that they differed by 1.2 ± 0.3 mm MDA and 6.9 ± 0.7 degrees over all patients. When the ASSET model was used to generate the 17 segments using each line, an average DSC of 0.89 ± 0.03 and an average MDA of 0.69 ± 0.29 mm was found which showed that the PCA-based method generates segmentations exceedingly close to the manually selected PLAX. This PCA-based method allows the ASSET model to be run by users who are not experienced in manually defining the PLAX.

Across the three experts, the average manual segmentation time to contour all 17 segments was 2.5 ± 0.5 h per patient. On average when each expert was compared to the ASSET model for the 10 patients, a DSC of 0.81 ± 0.06 and an average MDA of 0.99 ± 0.49 mm was found (Table 1). These values were comparable to the average DSC and MDA when comparing observers to one another, 0.83 ± 0.07 and 0.93 ± 0.47 mm, respectively (Table 1). A two-way analysis of variance (ANOVA) test revealed that no segment had a statistically significant difference in inter-observer variability (*p* > 0.05). On a scale of 1–5 (defined in Section 2.8), the patient’s segments were scored an average of 4.9 ± 0.2 across the 10 patients (Table 1). Three of the 10 patients received a single score of 4 (minor modifications required) and the rest of the scores (27/30) were a 5 (clinically acceptable as is).

### 3.3. Retrospective Dosimetric Assessment

**Figure 3:**
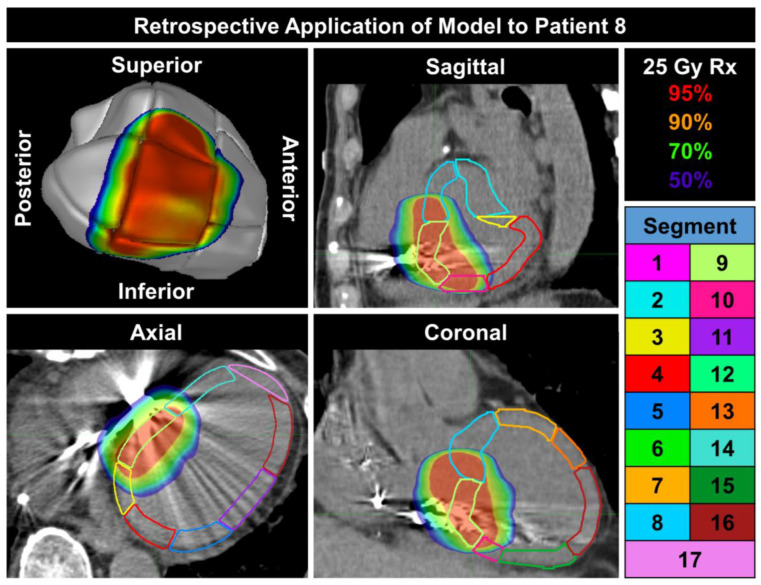
Retrospective application of ASSET for Patient 8 who was originally prescribed (Rx) 25 Gy to segment 9. The percentage of delivered dose is shown in the top right. The key outlining the display color for each LV segment is shown in the bottom right.

Patient records including information on which LV segments were included in the original VT target at time of treatment (derived from EAM data) were only tabulated at the time of original treatment for four patients. The other patients’ targets were not associated with a ventricular segment at time of treatment and instead a target was drawn manually by visual review of the EAM data. Figure 3 displays a retrospective dosimetric assessment of Patient 8 who was originally prescribed 25 Gy in 1 fraction to segment 9. The 17 LV segments have been overlaid onto the delivered dose. As seen in Figure 3, the 95% prescription dose line completely covers the automatically generated segment 9. Additionally, as seen in the sagittal and coronal views, the 95% prescription dose line extends further superiorly and inferiorly which is due to the added margin to account for patient cardiac and respiratory motion.

**Figure 4:**
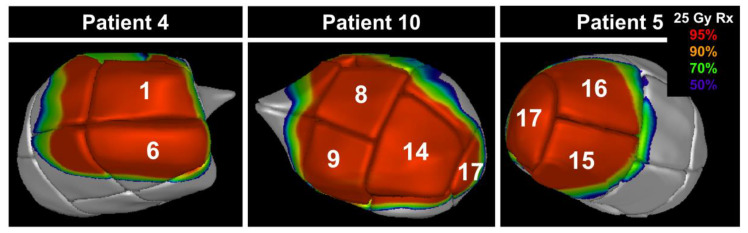
Three patient examples of ASSET applied retrospectively to various segments of the LVM for a 25 Gy prescription (Rx). The overlaid white number on each patient image represent the LV segments involved in the initial target for VT.

There were three other patients in the cohort that had target segment information in the patient treatment log which is displayed in Figure 4. For each of the three patients, the segments involved in the initial target for VT are highlighted in white. As can be seen for each patient, the involved segment, generated by the ASSET model, are covered by the 95% dose line with the overlay of the retrospective dose.

### 3.4. Prospective Patient Treatment

Since the development of the ASSET model, five patient treatments have been assisted though the use of this model. In total, the physician has on average saved 2 h per patient on target definition. Moreover, minimal corrections were required once the final treatment target was assembled by applying Boolean operations to the appropriate segments and a margin to generate the final planning target volume (PTV).

## 4. Discussion

To our knowledge, we are the first to present a semi-automated way to provide the AHA 17-segment model on CT treatment planning images for radiation therapy planning. The ASSET model was retrospectively applied to patients with CIEDs to allow for efficient and accurate segmentations. The derived PLAX through the use of PCA was shown to be clinically equivalent to the physician-defined PLAX. The developed ASSET model can be used on any imaging modality. Namely, there is much value to be obtained from using the ASSET model on three-dimensional MRI.

There have been consensus reports published showing how physicians prefer to interpret the AHA segmentation model [15] and suggested step-by-step approaches on how to implement it [16], which parallels the method described in this work. However, it should be noted that the AHA definitions of the 17 segments may be interpreted differently which could lead to differences in ground truth definitions across institutions. If a different interpretation of the AHA model is desired, the ASSET model can be quickly adapted to easily accommodate a different definition of ground truth.

Several studies have shown promising results when segmenting the cardiac substructures such as the ventricles, atria, great vessels, and coronary arteries on non-contrast-enhanced CT or MRI [21,22,23,24,25]. These studies have gone to great lengths to automatically segment the LV, for example, but have yet to create a way to automatically generate the 17 segments of the LV. As we did not want to introduce another measure of uncertainty, we did not include automatic LV segmentation in the current study. However, this would be the next logical step to include in the ASSET pipeline in the future and would allow for even further time-saving measures on the generation of the 17 segments of the LV. Notwithstanding, special measures would have to be taken in model training for an automatic LV segmentation to include a representative patient cohort for VT ablation (i.e., inclusion of stents, ICDs, ICPs, motion etc.). In the study by Morris et al. [22], DSC values > ~0.8 and MDA values < ~2.0 mm correlated to clinically acceptable segmentations regarding qualitative grading by expert physicians which parallels the experience from the current study. Additionally, in the study presented by Zhou et al. [25], inter-observer variability was conducted by eight experts and revealed MDAs near 2 mm for their best performing cardiac substructures (heart, aorta, atria, and ventricles) compared to <1 mm on average across all 17 segments of the LV for the three experts in the current study.

The major limitation of this work was the inability to verify manually segmented physician ground truth with EAM of the LV. In this study there were only four patients whose retrospective treatment data revealed which of the 17 segments of the LV was involved in the treatment target. The EAM data are garnered by cardiology to verify the presence of VT. If a registration could be achieved between this EAM data and the CT data used for radiation treatment planning, then the VT target could be drawn directly on the planning CT. Drawing directly on the CT data through the registration would also allow for the physician-drawn ground truth to be verified. However, this registration is difficult and associated with large registration errors up to a few millimeters due to several factors, including but not limited to, insufficient anatomical imaging data, large slice thicknesses for off-axis 2D cine images, and a different imaging co-ordinate system [26]. Furthermore, with a direct way of registering these datasets, the utility of the ASSET model would be lessened because the target could just be drawn directly. Several works, such as the one by Sharp et al. have shown that inter-observer variability can be used as a benchmark for accuracy evaluation [27]. In this work it was shown that the values for inter-observer variability were comparable to the values for the observers compared to the ASSET model giving confidence in the accuracy of the ASSET model. There are efforts being made to generate quality-assurance processes for fusing EAM data to radiotherapy planning data for cardiac radioablation such as this recent study presented by Mayinger et al. [28]. Although they were not able to complete a quantitative analysis of EAM target volume transfer, their results are promising (quality of transfer was evaluated on a limited cohort of less than five clinical cases) [28]. However, further validation of these methods is needed due to limited data availability and a lack of a full validation of the utilized software. Studies creating a way to ensure the quality of the data fusion process are useful to prevent major errors. Nevertheless, much improvement is still required to achieve a clinically acceptable level of uncertainty and accuracy, emphasizing the current value of manual target transfer procedures such as the ASSET model.

## 5. Conclusions

The novel model, ASSET, offers an efficient and precise way to automatically generate segmentations for the 17 segments of the LV on CTs for radiation therapy planning. As an aid for radiation therapy planning, ASSET offers significant time-saving measures and has strong potential for widespread adoption by institutions conducting radio-ablation of the LVM.

## 6. Patents

PCT patent application pending.

## Figures and Tables

**Table 1:** Average Dice similarity coefficient (DSC) and mean distance to agreement (MDA) between the three experts, as well as between the experts and the ASSET model for 10 patients. It also shows the median qualitative grade across three expert radiation oncologists along with the interquartile range (IQR). The qualitative grades are based on a scale (outlined in Section 2.8) with a maximum score of 5.

	Comparison Between Observers		Observers Compared with ASSET		
Patient	Average DSC	Average MDA (mm)	Average DSC	Average MDA (mm)	Median and IQR Qualitative Score
1	0.85 ± 0.07	0.83 ± 0.48	0.83 ± 0.07	0.95 ± 0.54	5.0 (5.0–5.0)
2	0.81 ± 0.09	1.16 ± 0.59	0.73 ± 0.06	1.33 ± 0.66	5.0 (5.0–5.0)
3	0.78 ± 0.09	1.45 ± 0.75	0.84 ± 0.04	0.87 ± 0.25	5.0 (4.3–5.0)
4	0.87 ± 0.04	0.62 ± 0.24	0.82 ± 0.07	0.99 ± 0.52	5.0 (5.0–5.0)
5	0.81 ± 0.09	1.08 ± 0.63	0.81 ± 0.07	1.04 ± 0.50	5.0 (5.0–5.0)
6	0.72 ± 0.11	1.68 ± 1.08	0.74 ± 0.11	1.63 ± 0.98	5.0 (5.0–5.0)
7	0.82 ± 0.06	0.76 ± 0.27	0.79 ± 0.08	1.03 ± 0.48	5.0 (5.0–5.0)
8	0.87 ± 0.07	0.57 ± 0.29	0.85 ± 0.07	0.66 ± 0.28	5.0 (4.3–5.0)
9	0.85 ± 0.07	0.62 ± 0.25	0.82 ± 0.07	0.83 ± 0.38	5.0 (4.3–5.0)
10	0.91 ± 0.03	0.51 ± 0.13	0.89 ± 0.06	0.60 ± 0.39	5.0 (5.0–5.0)
	**Average ± SD**				**Median and IQR**
**Average ± SD**	**0.83 ± 0.07**	**0.93 ± 0.47**	**0.81 ± 0.06**	**0.99 ± 0.49**	**5.0 (5.0–5.0)**

## Data Availability

The data presented in this study are not available in this article.
